# Homologous Cloning of Potassium Channel Genes From the Superior Apple Rootstock Line 12-2, Which is Tolerant to Apple Replant Disease

**DOI:** 10.3389/fgene.2022.803160

**Published:** 2022-01-26

**Authors:** Yunfei Mao, Yijun Yin, Xueli Cui, Haiyan Wang, XiaFei Su, Xin Qin, Yangbo Liu, Yanli Hu, Xiang Shen

**Affiliations:** National Key Laboratory of Crop Biology, College of Horticulture Science and Engineering, Shandong Agricultural University, Tai’an, China

**Keywords:** K^+^ channel genes, homologous molecular cloning, biochemical information, apple replant disease, ARD-resistant rootstock

## Abstract

Potassium channels are important ion channels that are responsible for the absorption of potassium in the plant nutrient uptake system. In this study, we used homologous molecular cloning to obtain 8 K^+^ channel genes from the superior apple rootstock line 12-2 (self-named): *MsAKT1-1*, *MsKAT3-2*, *MsKAT1-3*, *MsK2P3-4*, *MsK2P3-5*, *MsK2P5-6*, *MsK2P3-7*, and *MsK2P3-8*. Their lengths varied from 942 bp (*MsK2P5-6*) to 2625 bp (*MsAKT1-1*), and the number of encoded amino acids varied from 314 (*MsK2P5-6*) to 874 (*MsAKT1-1*). Subcellular localization predictions showed that *MsAKT1-1*, *MsKAT3-2*, and *MsKAT1-3* were localized on the plasma membrane, and *MsK2P3-4*, *MsK2P3-5*, *MsK2P5-6*, *MsK2P3-7*, and *MsK2P3-8* were localized on the vacuole and plasma membrane. The 8 K^+^ channel proteins contained α helices, extended strands, β turns, and random coils. *MsKAT1-3* had four transmembrane structures, *MsKAT3-2* had six, and the other six K^+^ channel genes had five. Protein structure domain analysis showed that *MsAKT1-1* contained nine protein domains, followed by *MsKAT3-2* with four, *MsKAT1-3* with three, and the other five two-pore domain K^+^ channel proteins with two. Semi-quantitative RT-PCR detection of the K^+^ channel genes showed that their expression levels were high in roots. qRT-PCR analysis showed that the relative expression levels of the 8 genes changed after exposure to ARD stress. The above results provide a theoretical basis for further research on the functions of potassium channel genes in 12-2 and a scientific basis for the breeding of ARD-resistant rootstock.

## Introduction

Apple replant disease (ARD) refers to the general phenomenon in which the same or closely related crops are continuously planted on the same piece of land, leading to yield reductions, quality deterioration, and impaired growth under normal management (Chen et al., 2020). Apple is grown throughout the world; many countries list apple as a major consumer product because of its strong ecological adaptability, high nutritional value, good storage characteristics, and long supply cycle (Mao et al., 2021c). Limited by land resources, the replanting of old orchards is becoming more and more common, and major apple-producing areas in the world are facing ARD (Mazzola and Manici, 2012). About 50% of the apple orchards in the United Kingdom, New Zealand, and Poland have ARD (Manici et al., 2013). ARD can lead to poor growth of new roots, slow growth, short plants, reduced resistance, disease, and death of the entire plant, leading to severe economic repercussions (Narwal 2010). Strategies for effectively ameliorating the effects of ARD on apple are urgently needed.

The ARD pathogen complex consists of oomycetes, including *Pythium* and *Phytophthora*, and fungi such as *Ilyonectria* and *Rhizoctonia*, at times acting in concert with the lesion nematode *Pratylenchus penetrans* (Zhu et al., 2016). However, the specific pathogen complex may vary across geographic regions, or even between orchards in the same region (Zhou et al., 2021). Previous research has shown that *Fusarium* is the main pathogen that causes ARD in the Bohai Bay area (Wang et al., 2018). The specialized *Fusarium proliferatum* strain MR5 (MW600437.1) associated with ARD has recently been screened and identified (in review); it is highly pathogenic to apple roots.

The development of resistant rootstocks has always been the focus of research for prevention and control of ARD. Leinfelder and Merwin (2006) documented the growth of four rootstocks (M26, M7, CG6210, and G30) after planting in replant soil for 4 years. The growth of the G30 and CG6210 plants increased significantly, and the average life span of the CG6210 root system was five times that of M7. Rumberger et al. (2004) grafted the Royal Empire variety onto three CG rootstocks (CG16, CG30, and CG210) and the M7 and M26 conventional rootstocks. Over three consecutive years, the CG210 and CG30 rootstocks were more resistant to ARD. Mazzola and Zhao (2010) reported that the rate of *Rhizopus* rot infection was significantly lower in the Geneva rootstock series than in M26, MM111, and MM166 in a Washington State replant orchard. Nevertheless, these rootstocks have not been promoted in China.

K^+^ is the most abundant key cation in almost all organisms, and it plays a key role in the basic physiological processes of plants (Adams and Shin, 2014). Two major types of plant potassium ion transporter genes have been cloned, potassium ion transporter genes and potassium ion channel genes (Flügge and Schroeder, 2007). Potassium channel genes mainly include the Shaker family and the *KCO* family. One of the important features of the Shaker potassium channels is that they can form heterotetrameric structures that enable plants to regulate potassium ion transport activity in different cells (Gambale and Uozumi, 2006). *AKT1* (Sentenac et al., 1992) and *KAT1* (Anderson et al., 1992) were the first cloned plant potassium channels, and both are members of the Shaker family. In 1997, Dreyer et al. (1997) cloned the potassium channel genes *AKT1*, *KAT1*, and *AtKAT3* in Arabidopsis. The potassium channel *SKOR* is expressed mainly in the central sheath and xylem parenchyma cells of Arabidopsis roots. It is responsible for the release of potassium ions from the column cells into the xylem and participates in potassium ion transport from the root to the crown (Gaymard et al., 1998). Czempinski was the first to find and clone the *TPK* family member *TPK1*/*KCO1* from Arabidopsis using a homologous alignment method (Czempinski et al., 1997), and six additional members (*TPK1*–*TPK5* and *KCO3*) were subsequently found in Arabidopsis (Czempinski et al., 1999). Two-pore domain potassium (*K2P*) channels are membrane proteins found in mammals, other animals (e.g., *Drosophila*), and multiple plant species (Goldstein et al., 2005; Enyedi and Czirják, 2010). Five genes encoding *K2P* channels have been found in the Arabidopsis genome (González et al., 2015).

K^+^ is also closely related to abiotic stress tolerance (Hasanuzzaman et al., 2018). K^+^ channels are ion channels responsible for the absorption of potassium in the plant nutrient uptake system. Xu et al. (2014) showed that the K^+^ channel gene *GhAKT1* could regulate the uptake of K^+^ and increase the stress resistance of cotton. Kobayashi et al. (2016) used genome-wide association analysis to show that *KATl* was closely related to salt tolerance in Arabidopsis. Li et al. (2020) demonstrated that the expression of the *KAT3* gene was induced by salt stress. A K^+^ uptake study of different apple rootstocks under ARD showed that the lower the ARD resistance, the more seriously the K^+^ absorption of the rootstocks was affected (Guo et al., 2015).

Previously, our research group selected and bred the new apple rootstock line 12-2 (self-named) through patented *in situ* breeding technology (Shen et al., 2015). A variety of physiological indexes were measured under ARD, and preliminary results showed that 12-2 had excellent resistance (Mao et al., 2021a). The use of non-damaging and non-destructive testing technology (Xu et al., 2020) to measure changes in K^+^ flow showed that ARD had little effect on K^+^ absorption in 12-2 (Mao et al., 2021b). Although numerous studies have shown that maintaining cell K^+^ balance is very important for plant resistance to various abiotic stresses (Assaha et al., 2017), there are few reports on how K^+^ channels affect plant resistance to ARD. In the present experiment, we used the K^+^ channel gene from Arabidopsis as a template and adopted a homologous cloning approach to identify K^+^ channel genes in 12-2. We then analyzed the relative expression of the K^+^ channel genes cloned in 12-2 under ARD-associated *Fusarium proliferatum* MR5 stress. Basic biochemical characterization of the multiple K^+^ channel genes and the results of quantitative reverse transcription-PCR (qRT-PCR) provide preliminary information on the molecular mechanisms by which K^+^ channel genes regulate root resistance to ARD in 12-2. They serve as an important theoretical basis for the breeding of ARD-resistant rootstocks.

## Materials and Methods

### Plant Materials and Experimental Treatments

The test material was the 12-2 rootstock that is tolerant to ARD and was selected through our patented *in situ* breeding technology. It was planted in the National Key Seedling Breeding Base of Shandong Agricultural University in Tai’an City, Shandong Province. At the beginning of May 2018, we selected sturdy twigs of 12-2 and cut them into 3-cm stem sections with new buds. After each section was rinsed with clean water for an hour, it was immersed in 75% alcohol for 30 s on an ultra-clean workbench that had been UV-sterilized for 30 min, then disinfected in 0.05% sodium hypochlorite solution for 10 min. It was washed three times with sterile water for 30 s each time. An oblique incision was made about 0.5 cm from the bottom of each stem segment with a sterilized scalpel, and the stems were inoculated on the induction medium. The medium was based on 1 L MS medium and contained 30 g L^−1^ sucrose (Sigma-Aldrich Co., Ltd., Shanghai, China), 7.5 g L^−1^ agar (Sigma-Aldrich), 0.6 mg L^−1^ 6-BA (Sigma-Aldrich), and 0.2 mg L^−1^ IBA (Sigma-Aldrich) at pH 5.8. Five buds were inoculated in each vial of induction medium and placed in a tissue culture chamber at 25 ± 2°C with a 10-h light period and an illumination intensity of 1000 lx. Substitution took place every 40 days on average. After multiple generations, at the beginning of May 2020, the adventitious bud clusters that had grown to about 4 cm were cut into about 2-cm stems and then inoculated into the rooting medium. The medium was based on 1 L 1/2 MS medium and contained 20 g L^−1^ sucrose, 7.5 g L^−1^ agar, 0.2 mg L^−1^ 6-BA, and 1.0 mg L^−1^ IBA at pH 5.8. After rooting, the collected roots were immediately frozen in liquid nitrogen and stored in a freezer at −80°C for RNA extraction. The extracted RNA was used for bioinformatics and semi-quantitative RT-PCR.

In mid-November 2021, one hundred 12-2 seedlings of similar size with 4–5 leaves were transplanted into black plastic pots (7.0 cm × 5.0 cm × 8.5 cm) filled with sterile substrate after seedling acclimatization. The specialized *Fusarium proliferatum* strain MR5 (MW600437.1) associated with ARD was recently screened and identified (in review); it is highly pathogenic to apple roots (Mao et al., 2021b) and was discovered by the research group of Professor Mao Zhiquan of Shandong Agricultural University. On November 24, a layer of pathogenic fungi was inoculated in liquid potato dextrose medium (PDB, Haibo, Qingdao, Shandong, China), cultured for 7 days, and then filtered through 8 layers of sterile gauze to obtain a spore suspension. The concentration was measured under a microscope (Nikon Ni-U, Tokyo, Japan) using a hemocytometer (Thermo Fisher Scientific, Waltham, MA, United States), and the final concentration was adjusted to 10^6^ spores mL^−1^ with sterile water. In early December, fifty pots of 12-2 were irrigated with 20 ml ARD-associated *Fusarium proliferatum* MR5 (MR5) spore suspension, and the other fifty pots were irrigated with an equal volume of PDB medium to serve as controls. The seedlings were grown in a tissue culture room at 24 ± 2°C with a 16-h light photoperiod and a light intensity of 1000 lx. The pots were bottom-irrigated as needed to maintain 60% soil water content. In early December, before irrigation with MR5 spore solution, the roots of the controls that had not been irrigated with PDB medium were sampled and recorded as MR5 spore solution treatment for 0 days. Samples were subsequently collected from both treatments at 1, 3, 5, 7, and 9 days (Mao et al., 2021b; Xiang et al., 2021), quick frozen in liquid nitrogen, and stored in a freezer at −80°C for use in RNA extraction. The RNA extracted at this time was used for qRT-PCR analysis. Three biological replicates of each treatment were obtained at each sampling time point.

### RNA Extraction and cDNA Synthesis

RNA was extracted from collected samples of 12-2 using the Invitrogen TRIzol Reagent kit (Thermo Fisher Scientific, Shanghai, China). The concentration and integrity of total RNA were determined by 0.8% agarose gel electrophoresis and a NanoDrop 2000 spectrophotometer (Thermo Fisher Scientific, Worcester, MA, United States). cDNA was synthesized by reverse transcription using the One-Step gDNA removal and cDNA synthesis kit (Transgen Biotech, Beijing, China).

### Cloning and Screening of K^+^ Channel Genes From 12-2

There is no reference genome for 12-2, and in order to identify its K^+^ channel genes, we used 19 K^+^ channel genes (8 *AKT*, 3 *KAT*, 6 *TPK*, and 2 *SKOR*) from the Arabidopsis (*Arabidopsis thaliana*) genome (https://www.arabidopsis.org/) as queries. We used BLASTP (Gallin and Boutet, 2011) to retrieve 28 K^+^ channel protein sequences from apple (*Malus domestica*) at NCBI (9 AKT, 4 KAT, 4 SKOR, and 11 two-pore domain potassium channel genes). Taking these 28 apple K^+^ protein channel sequences as templates, we used Primer Premier 6.0 (Wang et al., 2020) to design specific primers and ultimately cloned 8 K^+^ channel genes from 12-2 (Supplementary Table S1). With cDNA from 12-2 as a template, we used the high-fidelity enzyme 2 × Phanta Max Master Mix (Vazyme Biotech, Nanjing, Jiangsu, China) for PCR amplification. The 50-μL total reaction system contained 20 μL ddH2O, 1 μL cDNA template (500 ng μL^−1^), 2 μL upstream and downstream primers, and 25 μL high-fidelity enzyme. The amplification procedure was 98°C for 3 min; 35 cycles of 95°C for 30 s, 60°C for 30 s, and 72°C for 180 s; and 72°C for 10 min). A Gel Extraction Kit (CWBIO Biotech, Beijing, China) was used to recover bands of the appropriate lengths. These sequences were ligated into the p1300-GFP vector (Sangon Biotech, Shanghai, China). The resulting constructs were transformed into *E. coli* DH5α competent cells (Vazyme Biotech, Nanjing, Jiangsu, China), and the bacteria were cultured on LB solid medium containing kanamycin. The positive clones were screened by PCR, and the screened positive clones were sent to Shanghai Sangon Biotech Co., Ltd. for sequencing.

### K^+^ Channel Gene Searches of the Pear, Poplar, Rice, Tobacco, and Tomato Genomes

To explore the relationships between K^+^ channel genes in 12-2 and those from other species and to compare their physical and chemical properties, we used 19 K^+^ channel genes (8 *AKT*, 3 *KAT*, 6 *TPK*, and 2 *SKOR*) from Arabidopsis as BLASTP queries at NCBI. We retrieved 9 K^+^ channel protein sequences from pear (*Pyrus* spp*.*) (2 AKT, 4 KAT, and 3 SKOR), 13 from poplar (*Populus trichocarpa*) (6 *AKT*, 3 *KAT*, and 4 *SKOR*), 14 from rice (*Oryza sativa*) (7 *AKT*, 5 *KAT*, and 2 *SKOR*), 25 from tobacco (*Nicotiana tabacum* L) (8 *AKT*, 10 *KAT*, and 7 *SKOR*), and 14 from tomato (*Solanum lycopersicum*) (5 *AKT*, 6 *KAT*, and 3 *SKOR*).

### Multiple Sequence Alignment and Naming of K^+^ Channel Proteins in 12-2

DNAMAN software was used to construct a multiple sequence alignment of K^+^ channel proteins from 12-2 and apple, and the alignment results were combined with physical and chemical properties to predict the domain sequences and feature sites of each protein.

A phylogenetic tree was constructed from protein sequences of all species using MEGA7 software. The K^+^ channel proteins from 12-2 and the other species were grouped according to their cluster positions, and the K^+^ channel proteins from 12-2 were named according to the naming conventions of similar K^+^ channel proteins from apple.

### Sequence Analysis of K^+^ Channel Genes and Proteins in 12-2

The molecular weight, base composition, and base distribution of amino acid sequences were analyzed using DNAMAN software. The ORF Finder program (https://www.ncbi.nlm.nih.gov/orffinder/) was used to predict the open reading frames of the K^+^ channel genes in 12-2. The motif distributions within the K^+^ channel proteins were analyzed by MEME (Multiple Em for Motif Elicitation, http://meme-suite.org/tools/meme) (Bailey et al., 2015). The protein sequence file for 12-2 was submitted to MEME, with the number of motifs set to 10 and the length of motifs set to 6–50.

### Bioinformatics Analysis of K^+^ Channel Proteins in 12-2

ProtParam software (https://web.expasy.org/protparam/) (Garg, et al., 2016) was used to predict the isoelectric points, molecular masses, instability factors, fatty acid indexes, and GRAVY values of the 12-2 K^+^ channel proteins. Their subcellular localizations were predicted using ProtComp 9.0 (http://linux1.softberry.com/berry.phtml?topic=protcomppl&group=programs&subgroup=proloc) (Chou and Shen, 2010). DeepSig Server (https://deepsig.biocomp.unibo.it) (Savojardo et al., 2018) was used to predict the signal peptide sites of the proteins; their secondary structures, such as α helices, extended strands, β turns, and random coils, were predicted using PSIPRED (http://bioinf.cs.ucl.ac.uk/psipred/). The TMHMM Server v2.0 (http://www.cbs.dtu.dk/services/TMHMM/) (Krogh et al., 2001) was used to predict the transmembrane structure of the K^+^ channel proteins. The PFAM website (El-Gebali et al., 2019) was used to obtain positional information on K^+^ channel protein domains. The domains were imported into TBtools (Chen et al., 2018), and the Visualize Pfam Domain Pattern tool was used to visualize the protein domains.

### Semi-Quantitative RT-PCR Detection of K^+^ Channel Genes in Roots of 12-2

Using cDNA from 12-2 as a template, the 8 cloned potassium channel genes (Supplementary Table S2) were amplified using Taq Pro Universal SYBR qPCR Master Mix (Vazyme Biotech Co., Ltd., Nanjing, Jiangsu, China). The 20-μL reaction system contained 7 μL ddH_2_O, 1 μL cDNA template (500 ng μL^−1^), 1 μL upstream and downstream primers, and 10 μL Taq Pro Universal SYBR qPCR Master Mix. The amplification procedure was 95°C for 30 s; 40 cycles of 95°C for 10 s and 58°C for 30 s; 95°C for 15 s, 60°C for 1 min, and 95°C for 15 s. The *MdActin* (MD12G1140800) gene served as an internal control (Wang N. et al., 2015; Zhang et al., 2018). The amplification products were inspected and imaged using 1.0–1.5% agarose gel electrophoresis. ImageJ software (National Institutes of Health, United States) was used to analyze the gray values of the electrophoresis results. Three biological replicates of each sample were analyzed.

### Relative Expression of K^+^ Channel Genes in Roots of 12-2 After Exposure to ARD-Associated *Fusarium proliferatum* MR5

The qRT-PCR amplification reactions were performed using Taq Pro Universal SYBR qPCR Master Mix (Vazyme Biotech Co., Ltd., Nanjing, Jiangsu, China). The reaction system and temperatures were the same as those described in the preceding *Semi-Quantitative RT-PCR Detection of K* ^*+*^ *Channel Genes in Roots of 12-2*. Relative gene expression was normalized to that of the control treatment, and *MdActin* (MD12G1140800) served as the internal control (Wang N. et al., 2015; Zhang et al., 2018). The relative quantification of specific genes was performed using the cycle threshold (Ct) 2^−ΔΔCt^ method (SoftwareIQ5 version 2.0). Three biological replicates of each sample were analyzed. The qRT-PCR primers are listed in Supplementary Table S2.

## Results

### Cloning and Identification of K^+^ Channel Genes in 12-2

Using p1300-GFP as a vector for homologous cloning, 8 K^+^ channel genes were cloned based on 28 K^+^ channel gene sequences in apple (Table 1). Multiple sequence alignment (Supplementary Figures S1–S8) showed that the identity between the 8 K^+^ channel genes of 12-2 and their apple homologs ranged from 95.64 to 99.92%. This showed that the 8 K^+^ channel genes had been obtained from 12-2 by homologous cloning.

**TABLE 1:** Basic information on K^+^ channel genes from *Malus domestica*.

Gene ID in database	Gene length (bp)	CDS length (bp)	Gene ID in database	Gene length (bp)	CDS length (bp)	Gene ID in database	Gene length (bp)	CDS length (bp)
103405172	1688	1194	103444001	4944	2286	103407751	2396	1224
103405965	2105	945	103446212	3532	1293	103441938	7393	2610
103407916	2559	1059	103454313	5139	2673	114819255	1467	702
103415754	6681	2625	103456133	6534	1863	103444358	9038	2508
103417432	7251	2526	103456161	4892	2676	103444359	7231	1815
103423119	2481	789	103434967	3955	1290	103426610	6237	2646
103427530	4496	1062	103434415	1970	1086	103412399	5153	2316
103431252	5991	2292	103422957	1467	813	103441398	7596	1863
103432577	2645	774	114819988	756	756			
103443184	1904	1203	114819111	2982	1110			

**FIGURE 1:**
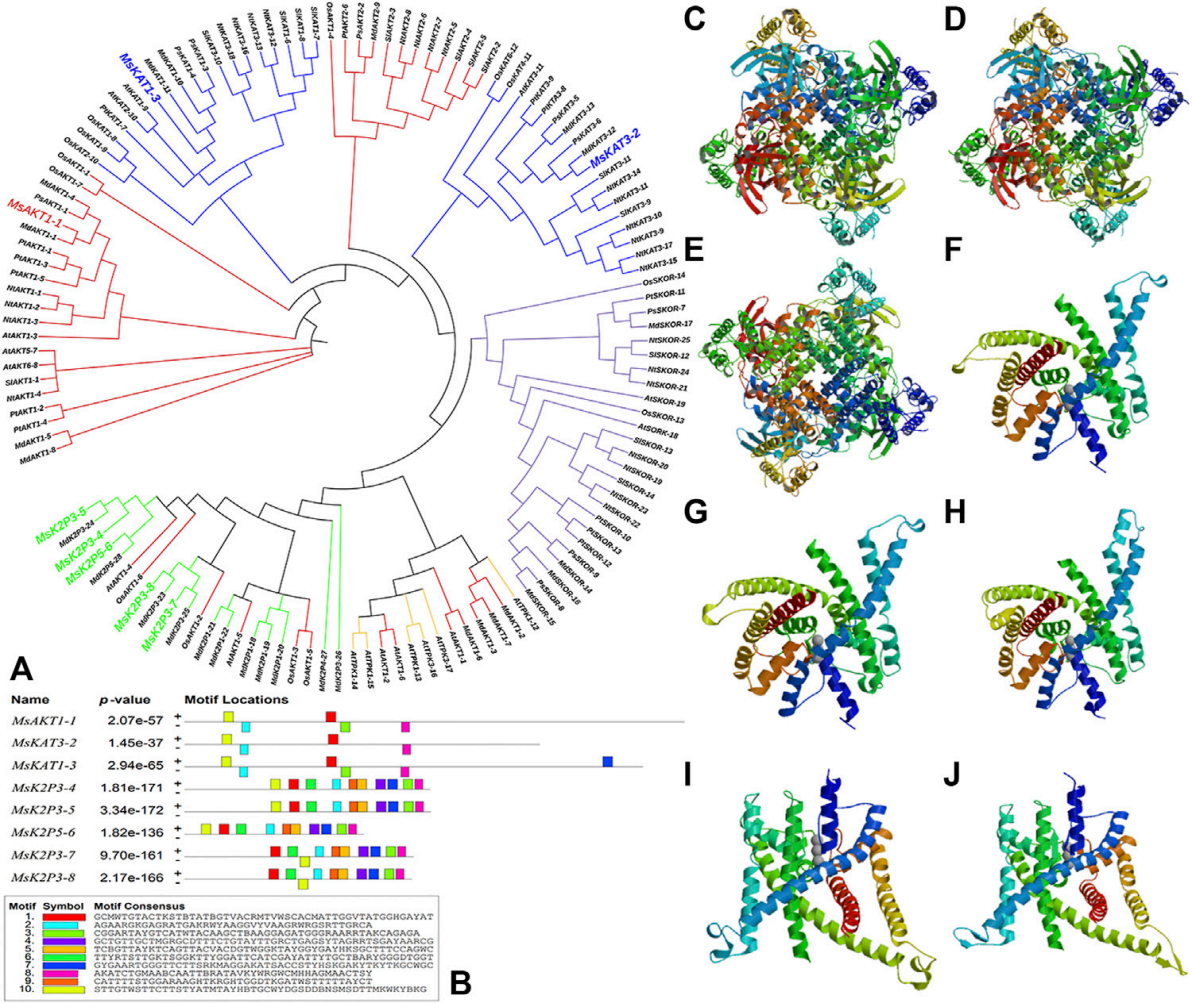
Phylogenetic tree of potassium channel genes in 12-2 and other species, conserved motifs in K^+^ channel genes, and secondary structure models of K^+^ channel proteins. **(A)**: phylogenetic tree of K^+^ channel genes in 12-2 and other species. Red, *AKT*. Blue, *KAT*. Purple, *SKOR*. Yellow, *TPK*. Green, two-pore domain K^+^ channel protein *(K2P)*. The 8 12-2 genes are enlarged and color-coded according to their respective family classifications. **(B)**: conserved motifs in K^+^ channel genes of 12-2. **(C–J)**: secondary structure models of K^+^ channel proteins in 12-2. **(C)**: *MsAKT1-1*. **(D)**: *MsKAT3-2*. **(E)**: *MsKAT1-3*. **(F)**: *MsK2P3-4*. **(G)**: *MsK2P3-5*. **(H)**: *MsK2P5-6*. **(I)**: *MsK2P3-7*. **(J)**: *MsK2P3-8*.

The phylogenetic tree (Figure 1A) showed that the 130 plant K^+^ channel proteins could be divided into 5 categories based on sequence homology. There were 46 *AKT* proteins (red in Figure 1A), 37 *KAT* proteins (blue), 25 *SKOR* proteins (purple), 6 *TPK* proteins (yellow), and 16 two-pore domain potassium (*K2P*) channel proteins (green). The K^+^ channel genes of 12-2 and other species all originated from one ancestor. The 8 genes from 12-2 were highly similar to those from apple, Arabidopsis, pear, and rice. The K^+^ channel genes from 12-2 were named according to their closest apple homologs using the format ‘Ms’ + K^+^ channel name + sequence name. Finally, the 8 genes (*MsAKT1-1*, *MsKAT3-2*, *MsKAT1-3*, *MsK2P3-4*, *MsK2P3-5*, *MsK2P5-6*, *MsK2P3-7*, and *MsK2P3-8*) were cloned from 12-2. The two-pore domain potassium (*K2P*) channel genes of 12-2 were closely related to some *AKT* genes from apple, Arabidopsis, and rice.

### Nucleotide Sequence Analysis of K^+^ Channel Genes in 12-2

Table 2 shows that the gene length and CDS length of the 8 K^+^ channels varied from 942 bp (*MsK2P5-6*) to 2625 bp (*MsAKT1-1*), the molecular weight of single-stranded DNA (ssDNA) varied from 290.84 kDa (*MsK2P5-6*) to 811.74 kDa (*MsAKT1-1*), the molecular weight of double-stranded DNA (dsDNA) varied from 580.75 kDa (*MsK2P5-6*) to 1618.21 kDa (*MsAKT1-1*), and the base compositions of the sequences also differed. Numbers of bases ranged from 205 (A in *MsK2P5-6*) to 717 (A in *MsKAT1-3*).

**TABLE 2:** Basic physicochemical properties of 8 K^+^ channel genes in 12-2.

Gene name	Gene length (bp)	CDS length (bp)	Molecular weight (kDa)		Composition				
			ssDNA	dsDNA	A	C	G	T	Other
*MsAKT1-1*	2625	2625	811.74	1618.21	708	574	639	704	0
*MsKAT3-2*	1866	1866	576.33	1150.28	550	412	412	492	0
*MsKAT1-3*	2407	1554	742.77	1483.77	717	546	511	633	0
*MsK2P3-4*	1290	1290	399.12	795.24	299	267	331	393	0
*MsK2P3-5*	1293	1293	400.00	797.08	305	268	328	392	0
*MsK2P5-6*	942	942	290.84	580.75	205	233	245	259	0
*MsK2P3-7*	1203	1203	371.70	741.61	320	276	290	317	0
*MsK2P3-8*	1194	1194	369.29	736.07	317	266	298	313	0

Transcription factor binding site predictions (Figure 1B) showed that there were five transcription factor binding sites in *MsAKT1-1*, four in *MsKAT3-2*, six in *MsKAT1-3*, and 10 in each of the five two-pore domain potassium channel proteins.

### Physicochemical Properties of K^+^ Channel Proteins

Table 3 shows that the number of amino acids in the 8 K^+^ channel proteins of 12-2 ranged from 314 (*MsK2P5-6*) to 874 (*MsAKT1-1*), with a minimum molecular weight of 35076.16 Da in *MsK2P5-6*, a maximum molecular weight of 97736.98 Da in *MsAKT1-1*, and an isoelectric point ranging from 7.3 (*MsAKT1-1*) to 8.97 (*MsK2P3-4*). The instability coefficients ranged from 20.72 (*MsK2P5-6*) to 48.16 (*MsKAT1-3*). *MsAKT1-1*, *MsK2P5-6*, *MsK2P3-7*, and *MsK2P3-8* were stable proteins (instability coefficients less than 40), whereas the other four were unstable proteins with fatty acid coefficients ranging from 94.86 (*MsKAT1-3*) to 116.34 (*MsK2P5-6*). Except for MsKT1-1 and *MsKAT3-2*, the other six proteins were hydrophobic.

**TABLE 3:** Physicochemical properties of K^+^ channel proteins.

Gene name	Number of amino acids	Molecular weight (Da)	Isoelectric point	Instability index	Aliphatic index	Grand average of hydropathicity
*MsAKT1-1*	874	97736.98	7.3	38.32	95.71	−0.116
*MsKAT3-2*	620	71861.31	8.15	41.34	99.81	−0.006
*MsKAT1-3*	518	60054.59	8.4	48.16	94.86	0.062
*MsK2P3-4*	429	47806.77	8.97	44.05	101.52	0.148
*MsK2P3-5*	430	47899.66	8.92	44.36	98.56	0.089
*MsK2P5-6*	314	35076.16	8.18	20.72	116.34	0.427
*MsK2P3-7*	400	44623.96	8.56	32.92	108.65	0.189
*MsK2P3-8*	397	44033.22	8.36	37.92	108.51	0.238

### Subcellular Localization of K^+^ Channel Proteins

Subcellular localization predictions (Table 4) indicated that three of the 8 K^+^ channels of 12-2 were localized to the plasma membrane (*MsAKT1-1*, *MsKAT3-2*, and *MsKAT1-3*), whereas the other five two-pore domain potassium channels were localized mainly to the vacuole and secondarily to the plasma membrane.

**TABLE 4:** Subcellular localization predictions for K^+^ channel proteins.

Gene name	Main position	Secondary position
*MsAKT1-1*	Plasma membrane	
*MsKAT3-2*	Plasma membrane	
*MsKAT1-3*	Plasma membrane	
*MsK2P3-4*	Vacuole	Plasma membrane
*MsK2P3-5*	Vacuole	Plasma membrane
*MsK2P5-6*	Vacuole	Plasma membrane
*MsK2P3-7*	Vacuole	Plasma membrane
*MsK2P3-8*	Vacuole	Plasma membrane

### Secondary Structures and Transmembrane Structures of K^+^ Channel Proteins

The DeepSig Server analysis showed that none of the 8 K^+^ channel proteins contained signal peptide sites. Secondary structure predictions (Table 5; Figures 1C–J) showed that the 8 proteins all contained α helices, extended strands, β turns, and random coils. The minimum percentage of amino acids in α helices was 41.65% (*MsAKT1-1*), and the maximum was 56.05% (*MsK2P5-6*); the percentage of amino acids in extended strands ranged from 9.79% (*MsK2P3-4*) to 19.25% (*MsK2P3-7*). The percentage of amino acids in β turns ranged from 2.56% (*MsK2P3-5*) to 7.78% (*MsAKT1-1*), and that in random coils ranged from 25.80% (*MsK2P5-6*) to 44.06% (*MsK2P3-4*). With the exception of *MsK2P3-4*, the amino acid percentages in the other seven proteins could be ranked α helix > random coil > extended strand > β turn. In all 8 proteins, the sum of the α helix and random coil percentages exceeded 75%.

**TABLE 5:** Secondary structure contents of K^+^ channel proteins in 12-2.

Gene name	α helix	Extended strand	β turn	Random coil	Number of amino acids
*MsAKT1-1*	364 (41.65%)	136 (15.56%)	68 (7.78%)	306 (35.01%)	874
*MsKAT3-2*	309 (49.84%)	103 (16.61%)	20 (3.23%)	188 (30.32%)	620
*MsKAT1-3*	259 (50.00%)	92 (17.76%)	24 (4.63%)	143 (27.61%)	518
*MsK2P3-4*	186 (43.36%)	42 (9.79%)	12 (2.80%)	189 (44.06%)	429
*MsK2P3-5*	188 (43.72%)	47 (10.93%)	11 (2.56%)	184 (42.79%)	430
*MsK2P5-6*	176 (56.05%)	43 (13.69%)	14 (4.46%)	81 (25.80%)	314
*MsK2P3-7*	186 (46.50%)	77 (19.25%)	16 (4.00%)	121 (30.25%)	400
*MsK2P3-8*	190 (47.86%)	54 (13.60%)	17 (4.28%)	136 (34.26%)	397

**FIGURE 2:**
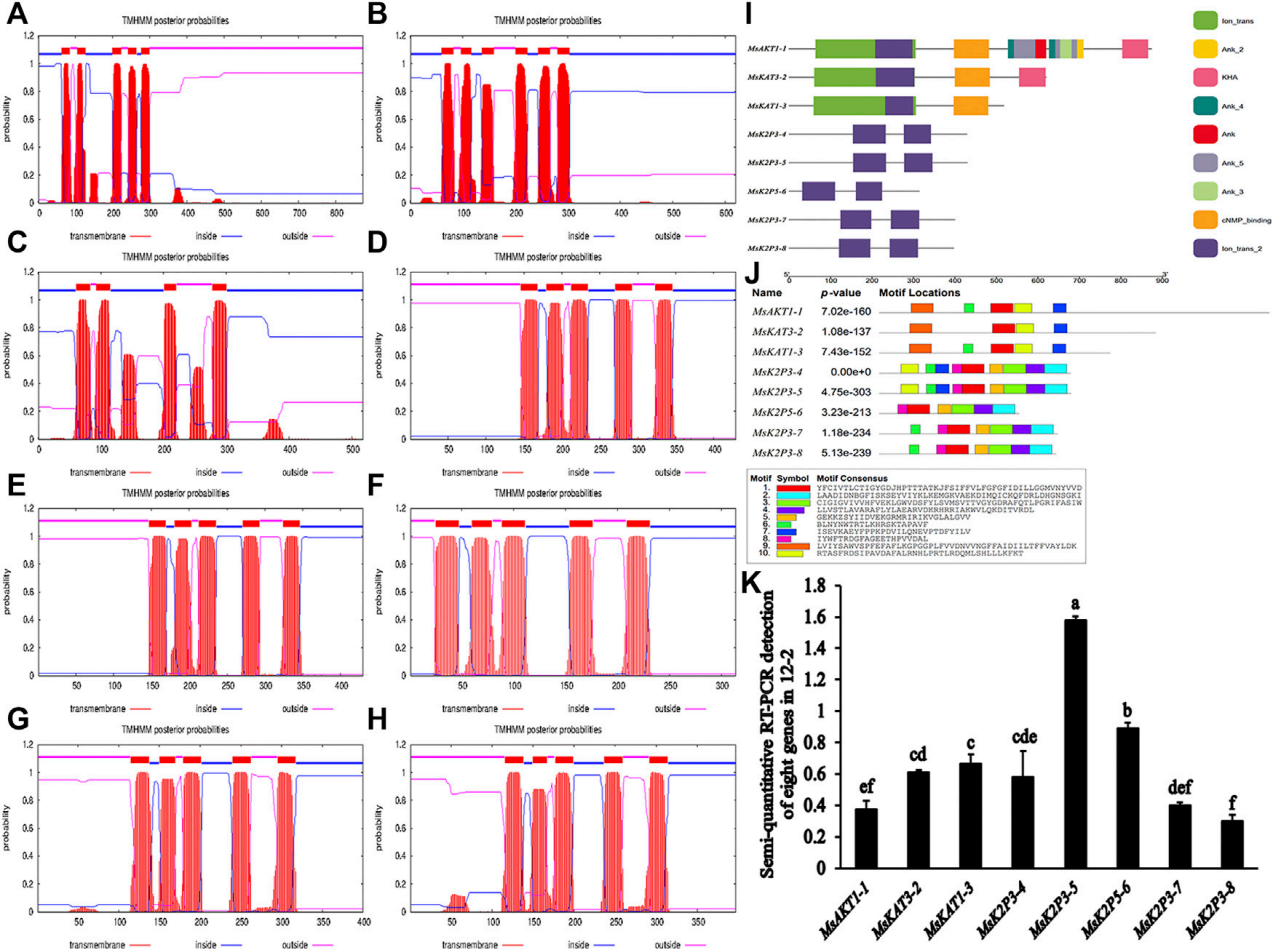
Models of transmembrane structures in K^+^ channel proteins, the structure of domains in K^+^ channel proteins, the motif distributions of K^+^ channel proteins, and semi-quantitative RT-PCR detection of 8 genes in roots of 12-2. **(A–H)**: models of transmembrane structures in K^+^ channel proteins of 12-2. **(A)**: *MsAKT1-1*. **(B)**: *MsKAT3-2*. **(C)**: *MsKAT1-3*. **(D)**: *MsK2P3-4*. **(E)**: *MsK2P3-5*. **(F)**: *MsK2P5-6*. **(G)**: *MsK2P3-7*. **(H)**: *MsK2P3-8*. **(I)**: the structure of domains in K^+^ channel proteins of 12-2. **(J)**: motif distributions of K^+^ channel proteins in 12-2. **(K)**: semi-quantitative RT-PCR detection of 8 genes in roots of 12-2.

Transmembrane structure predictions (Table 6; Figures 2A–H) showed that *MsKAT1-3* had four transmembrane structures, *MsKAT3-2* had six transmembrane structures, and the other six proteins had five transmembrane structures.

**TABLE 6:** Numbers of transmembrane structures and sequences of K^+^ channel proteins in 12-2.

Gene name	Number of transmembrane	Transmembrane sequences
*MsAKT1-1*	5	63–85, 105–127, 200–222, 242–264, 277–299
*MsKAT3-2*	6	60–82, 97–116, 137–159, 201–223, 244–266, 281–303
*MsKAT1-3*	4	61–83, 93–115, 201–220, 278–300
*MsK2P3-4*	5	146–168, 180–202, 212–234, 270–292, 323–345
*MsK2P3-5*	5	147–69, 181–203, 213–235, 271–293, 324–346
*MsK2P5-6*	5	25–47, 60–79, 89–111, 154–176, 209–231
*MsK2P3-7*	5	115–137, 150–169, 179–201, 240–262, 295–317
*MsK2P3-8*	5	116–138, 150–167, 177–199, 237–259, 292–314

### Protein Structures and Conserved Sites of K^+^ Channel Proteins

Protein domain analysis (Table 7; Figure 2I) showed that the number of domains was highest in *MsAKT1-1* (nine), followed by *MsKAT3-2* with four, *MsKAT1-3* with three, and the other five two-pore domain potassium channels with two. *MsAKT1-1*, *MsKAT3-2*, and *MsKAT1-3* were recognized as ion channel proteins, and the 8 proteins all contained Ion_trans_2 domains.

**TABLE 7:** The structure of domains in K^+^ channel proteins of 12-2.

Gene name	Hits found	Status	Identifier	Description
*MsAKT1-1*	9	DONE	Ion_trans	Ion transport protein
*MsKAT3-2*	4	DONE	Ion_trans	Ion transport protein
*MsKAT1-3*	3	DONE	Ion_trans	Ion transport protein
*MsK2P3-4*	2	DONE	Ion_trans_2	Ion channel
*MsK2P3-5*	2	DONE	Ion_trans_2	Ion channel
*MsK2P5-6*	2	DONE	Ion_trans_2	Ion channel
*MsK2P3-7*	2	DONE	Ion_trans_2	Ion channel
*MsK2P3-8*	2	DONE	Ion_trans_2	Ion channel

The distribution of conserved motifs (Figure 2J) showed that *MsAKT1-1* and *MsKAT1-3* contained five identical motifs, whereas *MsKAT3-2* contained four motifs. Compared with *MsAKT1-1*, *MsKAT3-2* lacked Motif 6. *MsK2P3-4* and *MsK2P3-5* both contained nine identical motifs (except Motif 9). *MsK2P3-7* and *MsK2P3-8* contained seven identical motifs. Compared with *MsK2P3-4*, they lacked Motif 7 and Motif 10. *MsK2P5-6* contained six motifs. Compared with *MsK2P3-7*, it lacked Motif 6. All 8 K^+^ channel proteins contained Motif 1.

### Semi-Quantitative RT-PCR Detection of 8 K^+^ Channel Genes in Roots of 12-2

To explore differences in expression level among the 8 K^+^ channel genes in roots of 12-2, we performed semi-quantitative RT-PCR detection of their expression levels by analyzing the brightness and gray levels of electrophoresis images (Figure 2K; Supplementary Figure S9). The expression levels of *MsK2P3-5* and *MsK2P5-6* were significantly higher than those of the other six genes, and the expression levels of the other six genes did not differ significantly.

### Relative Expression of K^+^ Channel Genes in Roots of 12-2 After Exposure to ARD-Associated *Fusarium Proliferatum* MR5

**FIGURE 3:**
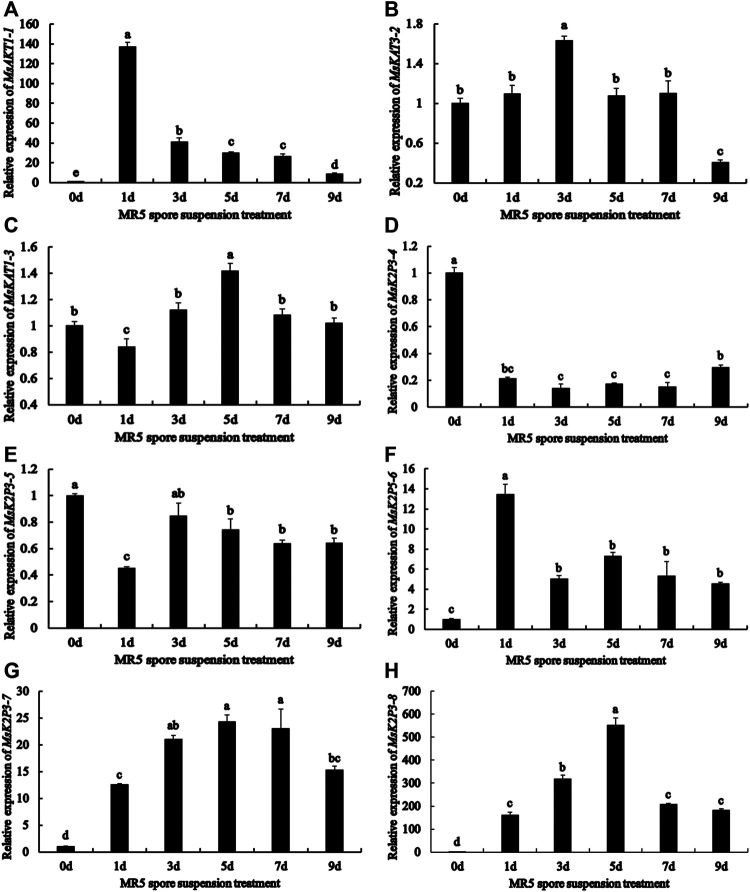
Relative expression of 8 K^+^ channel genes in roots at different days after spore solution treatment. **(A)**: *MsAKT1-1*. **(B)**: *MsKAT3-2*. **(C)**: *MsKAT1-3*. **(D)**: *MsK2P3-4*. **(E)**: *MsK2P3-5*. **(F)**: *MsK2P5-6*. **(G)**: *MsK2P3-7*. **(H)**: *MsK2P3-8*.

qRT-PCR analysis (Figure 3) showed that the relative expression of the 8 genes differed between roots treated with MR5 spore solution and control roots. Throughout the test period, the relative expression levels of *MsAKT1-1*, *MsK2P5-6*, *MsK2P3-7*, and *MsK2P3-8* were significantly higher in *Fusarium*-treated roots than in control roots. The relative expression of *MsAKT1-1* and *MsK2P5-6* was highest on the first day and then decreased with time. The relative expression of *MsK2P3-7* and *MsK2P3-8* first increased and then decreased, and their relative expression was highest on the fifth day. *MsKAT3-2*, *MsKAT1-3*, and *MsK2P3-5* showed a similar expression pattern: their expression first increased and then decreased. Relative expression of *MsKAT3-2* was highest on the third day and was significantly higher in *Fusarium*-treated roots than in control roots. Its expression was the lowest on the ninth day and was significantly lower in treated roots than in control roots. *MsKAT1-3* had the highest expression on the fifth day, when its expression was significantly higher in *Fusarium*-treated than control roots. The relative expression of *MsK2P3-5* peaked on the third day and there was no significant difference between *Fusarium*-treated and control roots. On the remaining days, its expression was significantly lower in *Fusarium*-treated than control roots. Throughout the experiment, the relative expression level of *MsK2P3-4* was significantly lower in treated than in control roots, and it showed a slowly increasing trend overall. Its relative expression was higher on the first day, then decreased and stabilized from the third to the seventh day. Its relative expression was highest on the ninth day and was significantly higher than that on the seventh day.

## Discussion

It has been suggested that there are two different types of K^+^ uptake systems in plants: the high-affinity K^+^ absorption system for external K^+^ concentrations of 0.001–0.2 mM and the low-affinity K^+^ absorption system for external K^+^ concentrations of 1–10 mM (Rai and Kawabata, 2020). The high-affinity K^+^ absorption system mainly involves K^+^ transporters, whereas the low-affinity system mainly involves K^+^ channel proteins (Xie et al., 2020). When plants are under stress and the amount of K^+^ is insufficient, the high-affinity K^+^ system is quickly activated, and plant high-affinity K^+^ absorption is mediated mainly by high-affinity K^+^ transporters (Shin and Schachtman, 2004; Schachtman and Shin, 2007).

In this study, we cloned 8 K^+^ channel genes from the 12-2 rootstock through homologous cloning. Phylogenetic analysis showed that the K^+^ channel genes in 12-2 had high similarity to those from apple. This reflects not only the differentiation of monocots and dicots during plant evolution (Parkin, 1926) but also the fact that 12-2 and apple are members of the same genus. Research by Shealy et al. (2003) showed that the Shaker family contained the highly conserved amino acid sequence TxxTxGYGD (the T box). A TMCTIGYGD sequence was present at amino acids 191–199, 192–200, and 69–77 in *MsK2P3-4*, *MsK2P3-5*, and *MsK2P5-6*, respectively, and a TLCTIGYGD sequence was present at amino acids 159–167 and 156–164 in *MsK2P3-7* and *MsK2P3-8*. The five two-pore domain potassium (*K2P*) channel genes therefore all contained T box structures. This may be one of the reasons why two-pore domain potassium (*K2P*) channel genes of 12-2 were highly similar to some *AKT* genes of apple, Arabidopsis, and rice in this research.

The results showed that the gene lengths and CDS lengths of the 8 K^+^ channel genes, and the number of translatable amino acids, unlike other species such as Arabidopsis. These results were in agreement with the study of Moulton et al. (2003), which showed that the number of amino acids differed among potassium channel proteins from distantly related species, although there was also conservation among species. The differences in gene length and CDS length also caused differences in the molecular weight of single-stranded DNA and double-stranded DNA, the base compositions of the nucleotide sequences, and the transcription factor binding sites. The numbers of amino acids also led to differences in molecular weight, isoelectric point, instability index, aliphatic index, and grand average of hydropathicity.

Studies have shown that *AKT1* proteins are located on the plasma membrane (Cuéllar et al., 2013), consistent with the localization prediction for *MsAKT1-1* in this study. Amino acids 260–268 of *MsKAT3-2* were TLTTVGYGD, indicating that *MsKAT3-2* contained a T-box and belonged to the Shaker gene family. *KC1* (*KAT3*) family genes are mainly expressed in root hairs and endothelial cells, and they participate in the regulation of K^+^ absorption by the *AKT1* channel (Ragel et al., 2019). Jin et al. (2021) showed that members of the Shaker family were all located on the plasma membrane and were all highly conserved. The five two-pore domain potassium (*K2P*) channels in 12-2 were predicted to be located on the vacuole and plasma membrane, consistent with the results of Voelker et al. (2010) on the subcellular localization of *K2P* channels in Arabidopsis.

The results of secondary structure in this research were in agreement with those of Gao et al. (2021), and the 8 K^+^ channel proteins all contained multiple transmembrane structures. *MsAKT1-1* channel proteins had the five transmembrane structures typical of Shaker proteins (Yang et al., 2019), and amino acids 63–299 were the K^+^ transport region. This was in contrast to a study of *AKT1* channel proteins in soybean by Wang Y. Q. et al. (2015). The number of transmembrane structures of the other seven K^+^ channel genes were also different. These results might also be related to the diversity of expanded membrane structures in the Shaker family (Jin et al., 2021). The results of protein domain analysis further verified their identity as potassium channel proteins. Conserved motif analysis showed that the number of conserved amino acid sites was positively correlated with the number of nucleotide transcription factor binding sites (Yura and Go, 2008). The 8 K^+^ channel proteins all contained Motif 1, and Motif 1 contained a T box, providing further evidence that the *K2P* channel genes of 12-2 were highly similar to some *AKT* genes from apple, Arabidopsis, and rice.

Semi-quantitative RT-PCR detection and qRT-PCR analysis showed that the 8 potassium channel genes may participate in rhizosphere K^+^ homeostasis of 12-2 by responding to ARD stress. These results also help to explain our previous findings on ion currents in 12-2 roots (Mao et al., 2021b). A study by Fuchs et al. (2005) showed that the K^+^ channel gene *OsAKT1* regulated the absorption of K^+^ to improve rice salt tolerance, and a study by Jin et al. (2017) showed that ion channels could increase plant drought resistance through K^+^ efflux. *KAT1* genes encode plant inward K^+^ channels (Philippar et al., 2003) and are closely related to plant abiotic stress tolerance (Hasanuzzaman et al., 2018). Guo et al. (2021) found that *KAT* genes were related to drought resistance. *K2P* channel proteins have been found in humans and mammals and are located on membranes (Goldstein et al., 2005). Five genes encoding *K2Ps* have been found in the Arabidopsis genome and are involved in a variety of physiological functions, such as cell volume regulation and apoptosis (Nematian-Ardestani et al., 2020). Although no studies have shown a direct relationship between *K2P* genes and resistance, these genes have a marked effect on vacuole homeostasis (Kintzer and Stroud, 2016). Feng et al. (2021) showed that a vacuole gene was closely related to stress resistance in cotton.

## Conclusion

8 K+ channel genes were cloned from the superior apple rootstock line 12-2 with improved ARD resistance by homologous cloning. Basic biochemical characterization, semi-quantitative RT-PCR, and qRT-PCR showed that the 8 potassium channel genes were associated with ARD resistance and played an important role in maintaining the rhizosphere K+ homeostasis of 12-2. The above results provide a theoretical basis for further research on the functions of potassium channel genes in 12-2 and also provide a scientific basis for the breeding of ARD-resistant rootstock.

## Data Availability

The data that support the findings of this study are available on request from the corresponding author, and have been submitted to GenBank (GenBank accession numbers for 8 genes are OL742694, OL742695, OL742696, OL742697, OL742698, OL742699, OL742700, and OL742701). Nucleotide alignments from this article are listed in Additional file 1 (Supplementary Figures S1–S8).

## Abbreviations

12-2, superior apple rootstock line named 12-2; ARD, apple replant disease; MR5, ARD-associated Fusarium proliferatum MR5; K2P, two-pore domain potassium.
