# Supplementary material for: Homologous Cloning of Potassium Channel Genes From the Superior Apple Rootstock Line 12-2, Which is Tolerant to Apple Replant Disease
Source: Front Genet. 2022 Jan 26;13:803160. doi: 10.3389/fgene.2022.803160 (PMC8826240; doi:10.3389/fgene.2022.803160)
Supplement: Supplementary file 1 [file DataSheet1.PDF]

Additional file 1:

Supplementary Table S1. Primer sequences for cloning eight K^+ channel genes

Gene name	Forward primers (5′-3′)	Reverse primers (5′-3′)
MdAKT1-like	ATGGAGAGCAATAGCAGCACCA	TTGTTGTGATTCACCAGCACTTACA
MdKAT3	ATGTCGTCGTGGTCGGCA	AAAGATAAACAAGCGGTCGTTGT
MdKAT1-like	ATGACGTTTTCATGCACGAAAA	AGCCGCATCAGAATTCATATTATTA
MdK2P3	ATGGAGAAAGAGCCATTACTCCC	AGGTCGACTCAAAAGATCAGCAA
MdK2P3-like	ATGGAGAAAGAGCCGTTGCTG	ATGTCGACTCAAGAGATCAGCAAG
MdK2P5	ATGGCCGTTATGCAAGACCTC	CAAGCGATGCGCCAACAG
MdK2P3-like	ATGGACGAACCTTTACTTTCCAGG	CATGTCGCTTCCCATTAGATCA
MdK2P3-like	ATGGACGAACCTTTTATTTCCAGG	CATGTTGCTTCCCATTATATCAACA

Supplementary Table S2. Primer sequences for the semi-quantitative and qRT-PCR validation of eight K^+ channel genes

Gene name	Forward primers (5′-3′)	Reverse primers (5′-3′)
MdAKT1-like	GCGAAAACATGGATGGGAGC	TCCCGTGCATTGACTGGATG
MdKAT3	GGATGGCAACCCATCACAAAC	TTCTCTCCGAAGTTGACCGC
MdKAT1-like	CGGACAACCGAACTTTCCCA	GAAGGGTGGTGTGCATGACT
MdK2P3	GCAGGATTCTCGTCCTCTCG	GGGGTCGATGAGCCATGATT
MdK2P3-like	GTCTTTACCAGGCCGCATCT	ACCCTAGCCTCAGCCAAGTA
MdK2P5	GTTCTGTGCATCGGCATTGG	CGAAAACAGAAGCCACACGG
MdK2P3-like	AACAAGCGGAGACAGCTCAA	AGCTTGGCGAACGATGAGAG
MdK2P3-like	ACACCCTTCATTGTTCGCCA	GTGAAGAGCTTGGTAGCCGT
MdActin	TGACCGAATGAGCAAGGAAATTACT	TACTCAGCTTTGGCAATCCACATC

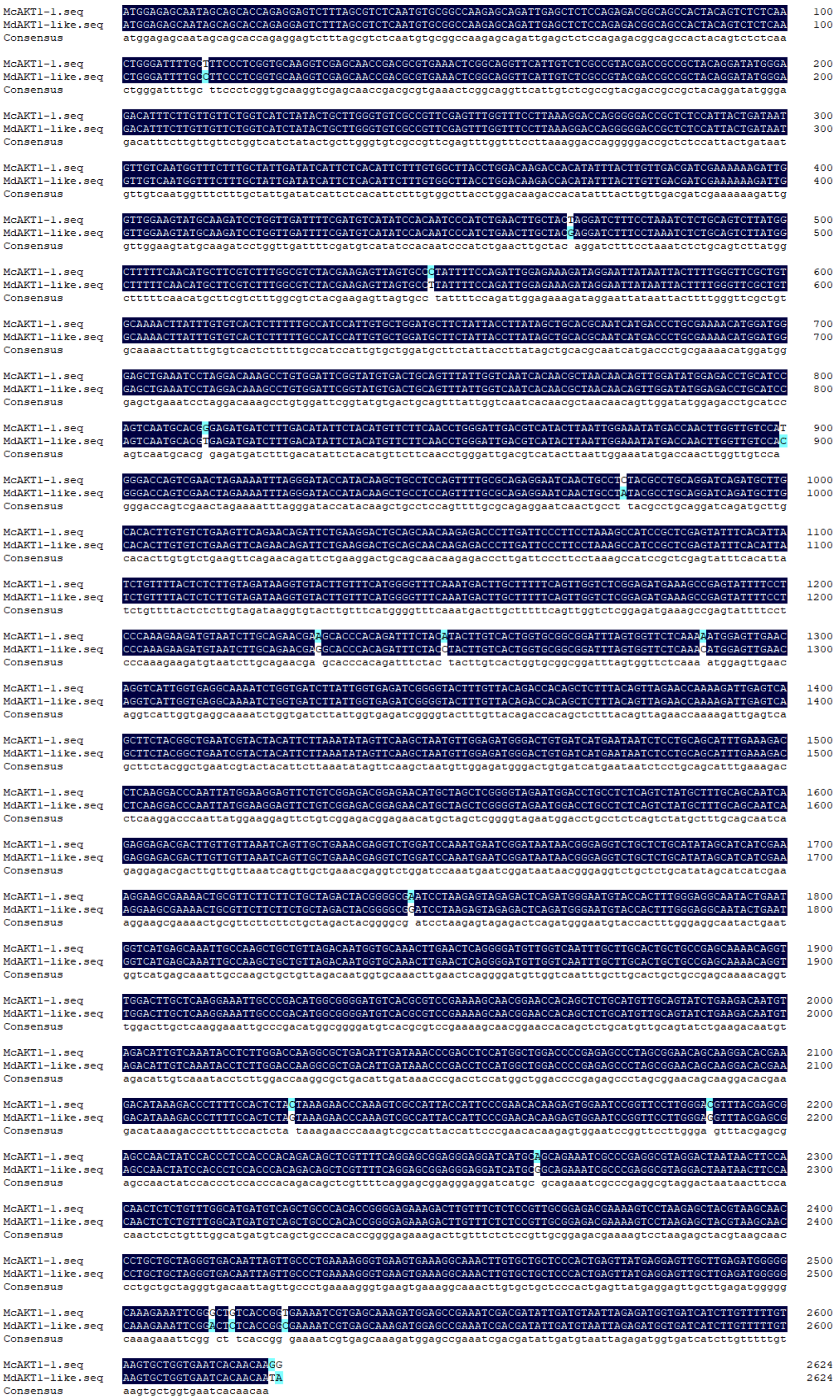

Supplementary Figure S1. Nucleotide alignments of *MsAKT1-1* with *MdAKT1-like* from NCBI. Comparison of nucleotide of *MsAKT1-1* we cloned and *MdAKT1-like* (LOC103415754) downloaded from NCBI. The similarity was 99.31%. NCBI, National Center for Biotechnology Information.

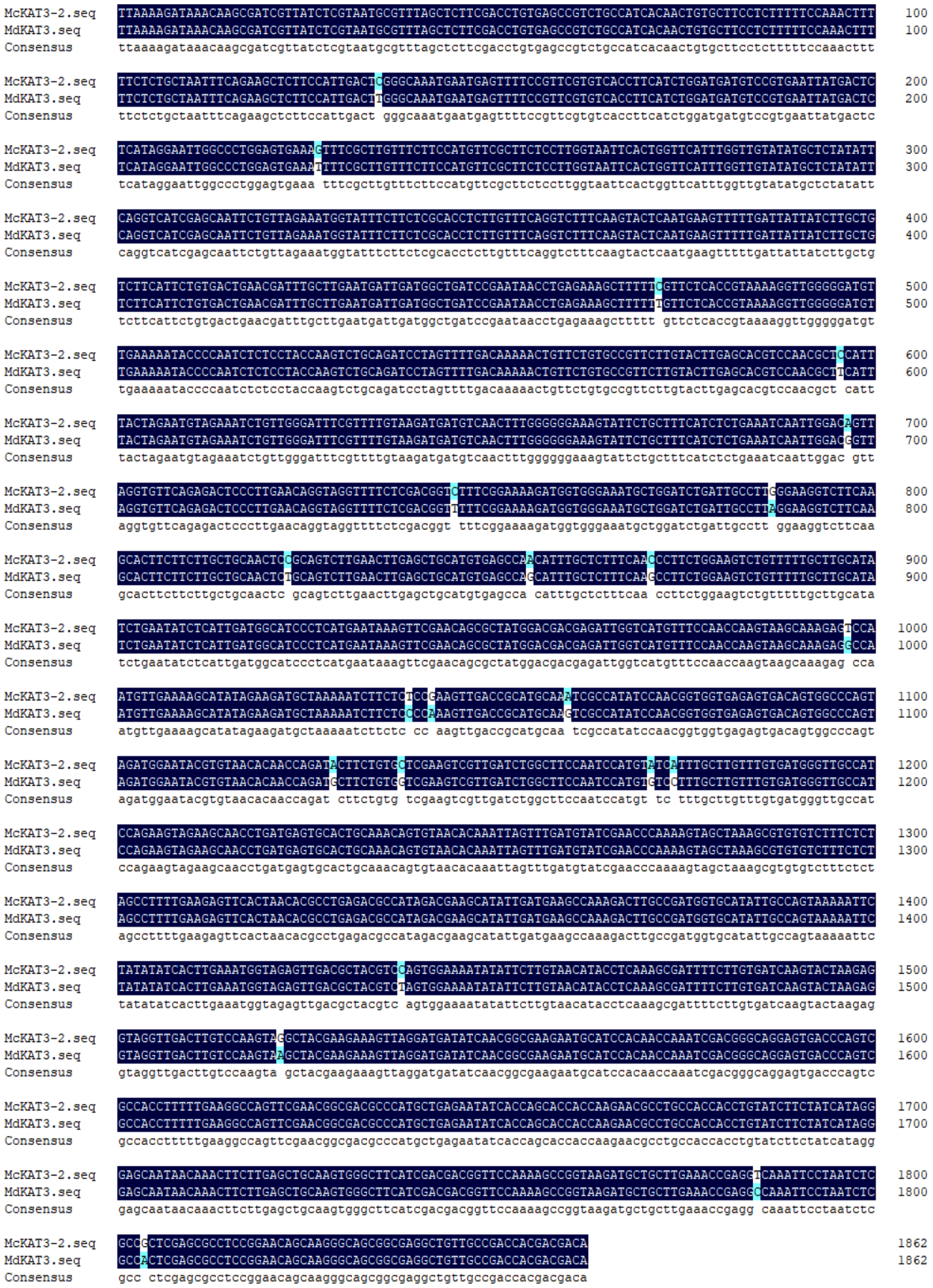

Supplementary Figure S2. Nucleotide alignments of *MsKAT3-2* with *MdKAT3* from NCBI. Comparison of nucleotide of *MsKAT3-2* we cloned and *MdKAT3* (LOC103456133) downloaded from NCBI. The similarity was 98.82%. NCBI, National Center for Biotechnology Information.

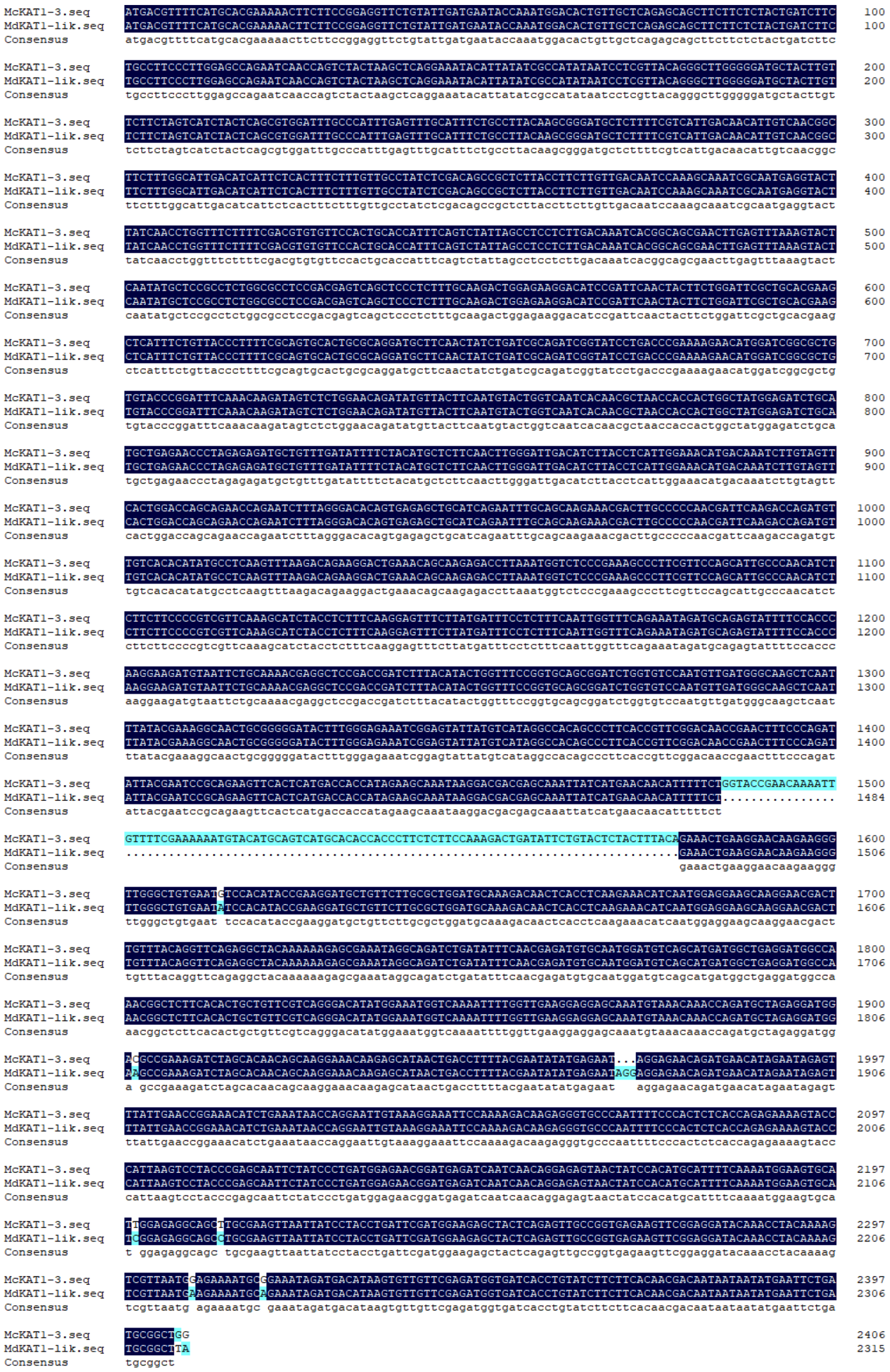

Supplementary Figure S3. Nucleotide alignments of *MsKAT1-3* with *MdKAT1-like* from NCBI. Comparison of nucleotide of *MsKAT1-3* we cloned and *MdKAT1-like* (LOC103412399) downloaded from NCBI. The similarity was 95.64%. NCBI, National Center for Biotechnology Information.

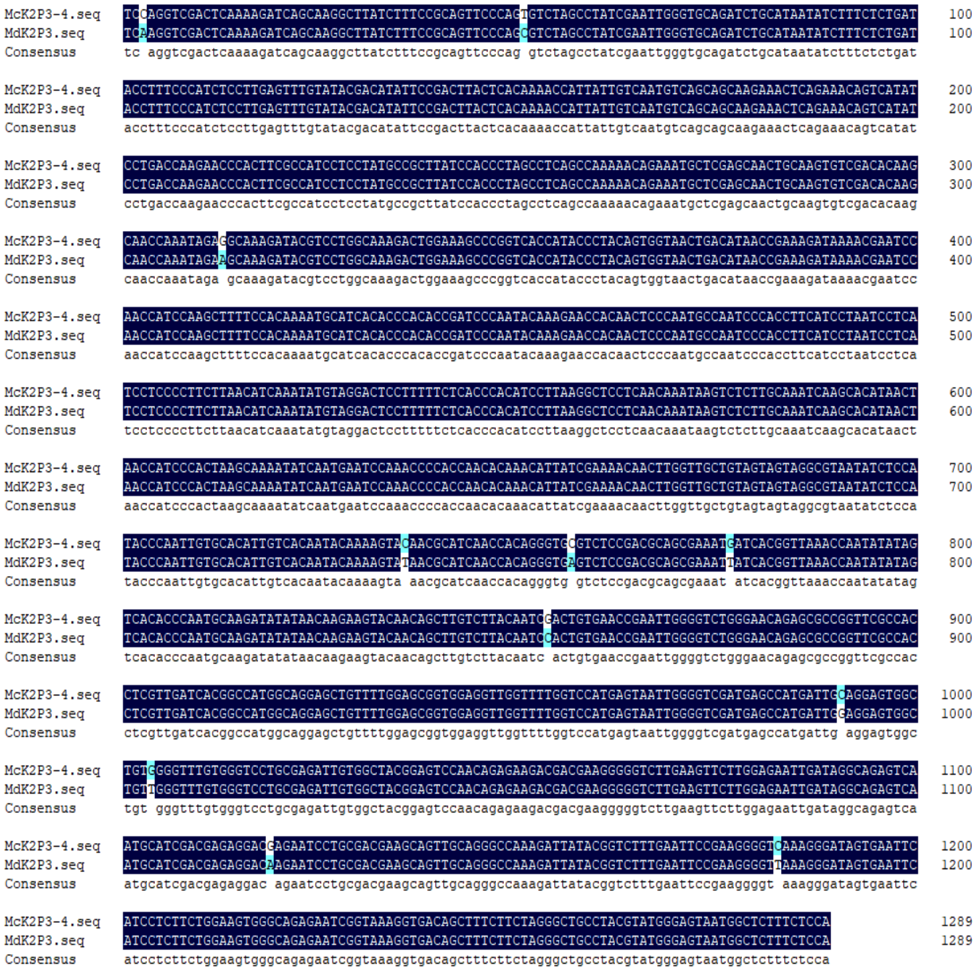

Supplementary Figure S4. Nucleotide alignments of *MsK2P3-4* with *MdK2P3* from NCBI. Comparison of nucleotide of *MsK2P3-4* we cloned and *MdK2P3* (LOC103434967) downloaded from NCBI. The similarity was 99.15%. NCBI, National Center for Biotechnology Information.

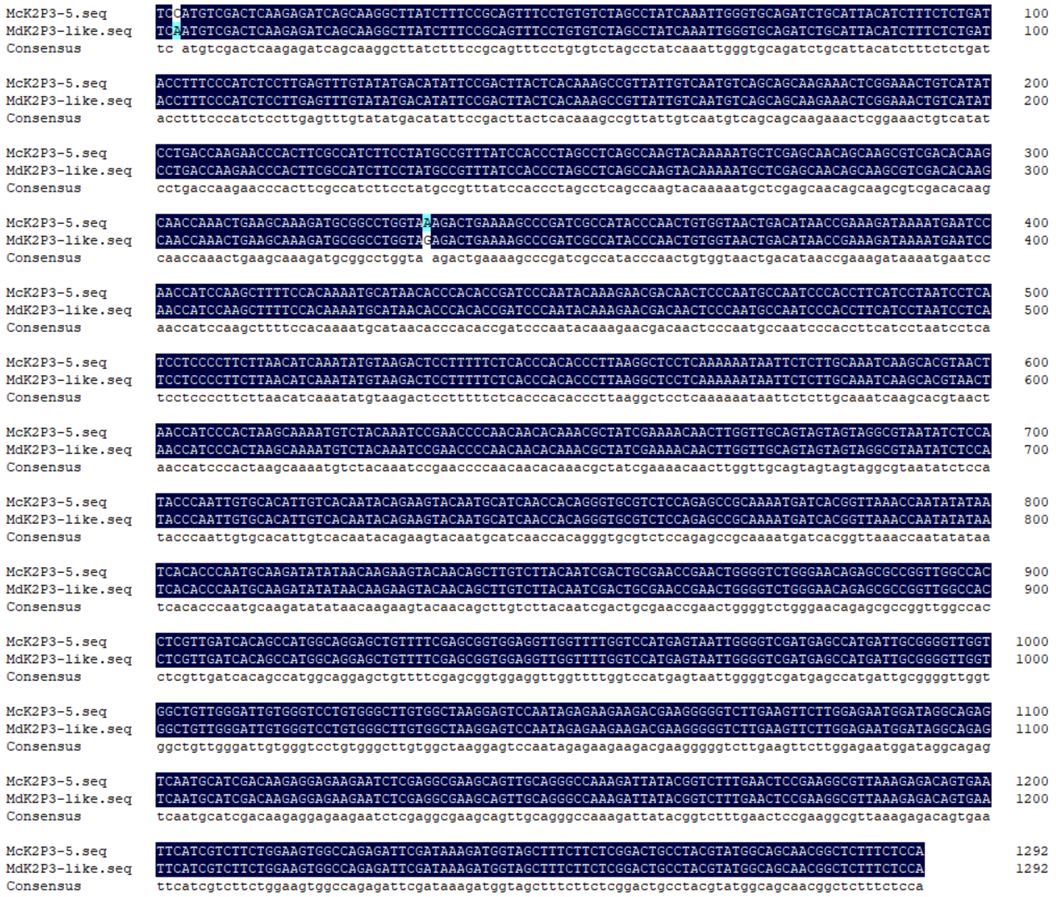

Supplementary Figure S5. Nucleotide alignments of *MsK2P3-5* with *MdK2P3-like* from NCBI. Comparison of nucleotide of *MsK2P3-5* we cloned and *MdK2P3-like* (LOC103446212) downloaded from NCBI. The similarity was 99.85%. NCBI, National Center for Biotechnology Information.

```
McK2P5-6.seq   TCCCAAGCGATGCGCCAACAGATCAGGCAGTGTTATCTTCCCAGAGTGGTTCTGGTCAAGCCTACTGAACTGATCGCAGATTTGCAGAATATCTTTCTCC   100
MdK2P5.seq     TTACAAGCGATGCGCCAACAGATCAGGCAGTGTTATCTTCCCAGAGTGGTTCTGGTCAAGCCTACTGAACTGATCGCAGATTTGCAGAATATCTTTCTCC   100
Consensus      t  caagcgatgcgccaacagatcaggcagtgttatcttcccagagtggttctggtcaagcctactgaactgatcgcagatttgcagaatatctttctcc

McK2P5-6.seq   CCTATCTTTCCCATCTCTTTAAGCTTGTAAATAACATACTCTGACTTACTGATGAAACCATGGTTATTGATGTCGGCGGCAAGCAGATCCTGGACGGTGA   200
MdK2P5.seq     CCTATCTTTCCCATCTCTTTAAGCTTGTAAATAACATACTCTGACTTACTGATGAAACCATGGTTATTGATGTCGGCGGCAAGCAGATCCTGGACGGTGA   200
Consensus      cctatctttcccatctctttaagcttgtaaataacatactctgacttactgatgaaaccatggttattgatgtcggcggcaagcagatcctggacggtga

McK2P5-6.seq   TATCCCTATGCAAAACCCAGTTCATAATCCTCCTGTGCCTTTTGTCCACCCTGGCCTCTGCTAAATATATAAACGCCCGAGCCACCGCCAACGTCGAAAA   300
MdK2P5.seq     TATCCCTATGCAAAACCCAGTTCATAATCCTCCTGTGCCTTTTGTCCACCCTGGCCTCTGCTAAATATATAAACGCCCGAGCCACCGCCAACGTCGAAAA   300
Consensus      tatccctatgcaaaacccagttcataatcctcctgtgccttttgtccaccctggcctctgctaaatatataaacgcccgagccaccgccaacgtcgaaaa

McK2P5-6.seq   CAGAAGCCACACGGCGGCGAAGAGTCTCCCTTGGAGAGTCTGGAAGGCCCTGTCACCGTACCCAACCGTCGTAACCGACATAACGGACAAGTAAACAGAA   400
MdK2P5.seq     CAGAAGCCACACGGCGGCGAAGAGTCTCCCTTGGAGAGTCTGGAAGGCCCTGTCACCGTATCCAACCGTCGTAACCGACATAACGGACAAGTAAACAGAA   400
Consensus      cagaagccacacggcggcgaagagtctcccttggagagtctggaaggccctgtcaccgta ccaaccgtcgtaaccgacataacggacaagtaaacagaa

McK2P5-6.seq   TCAATCCAGTCCAAATTCTCTACAAAACATAACACCAAAGCCCCAATGCCGATGCACAGAACCACCACCCCAAGCGCCAGGCCGACCTTGAGTCGGATTC   500
MdK2P5.seq     TCAATCCAGTCCAAATTCTCTACAAAACATAACACCAAAGCCCCAATGCCGATGCACAGAACCACCACCCCAAGCGCCAGGCCGACCTTGAGTCGGATTC   500
Consensus      tcaatccagtccaaattctctacaaaacataacaccaaagccccaatgccgatgcacagaaccaccaccccaagcgccaggccgaccttgagtcggattc

McK2P5-6.seq   TCATCCTCCCCTTTGCCACATCGACAATATAGTCCCTGGCGGAGAAGCCATGGTTCCGGGAAGTCTGACCCATTTGAATTCCGGTCAAGATCATGTTTTC   600
MdK2P5.seq     TCATCCTCCCCTTTGCCACATCGACAATATAGTCCCTGGCGGAGAAGCCATGATTCCGGGAAGTCTGACCCATTTGAATTCCGGTCAAGATCATGTTTTC   600
Consensus      tcatcctcccctttgccacatcgacaatatagtccctggcggagaagccatg ttccgggaagtctgacccatttgaattccggtcaagatcatgttttc

McK2P5-6.seq   TTGCAAGTCGAGGACAAAATTGACGACCCCACTGAGCAAAATGTCGATGAAACCGAACCCGAAGAGCACAAAGACGCAAGCGAAAATCTTGGTGAGAGGA   700
MdK2P5.seq     TTGCAAGTCGAGGACAAAATTGACGACCCCACTGAGCAAAATGTCGATGAAACCGAACCCGAAGAGCACAAAGACGCAAGCGAAAATCTTGGTGAGAGGA   700
Consensus      ttgcaagtcgaggacaaaattgacgaccccactgagcaaaatgtcgatgaaaccgaacccgaagagcacaaagacgcaagcgaaaatcttggtgagagga

McK2P5-6.seq   GTGGTGGGAGCAATGTCACCGTACCCAATGGTGCACATGGTGACTATACAGAAGTAGAGGGCGTCGACGACCGGGTGGGTTTCGACGCCGGAGAATTTGT   800
MdK2P5.seq     GTGGTGGGAGCAATGTCACCGTACCCAATGGTGCACATGGTGACTATACAGAAGTAGAGGGCGTCGACGACCGGGTGGGTTTCGACGCCGGAGAATTTGT   800
Consensus      gtggtgggagcaatgtcaccgtacccaatggtgcacatggtgactatacagaagtagagggcgtcgacgaccgggtgggtttcgacgccggagaatttgt

McK2P5-6.seq   CCCTGCTGAAGGAGTAAATTACAACGCCGAGCGATAGGTAGACGAGGAGCAAGAAGACGGCTTGCCTGATGATGGAGGTGGAGTCCGATTGTGGTTTGGG   900
MdK2P5.seq     CCCTGCTGAAGGAGTAAATTACAACGCCGAGCGATAGGTAGACGAGGAGCAAGAAGACGGCTTGCCTGATGATGGAGGTGGAGTCGGATTGTGGTTTGGG   900
Consensus      ccctgctgaaggagtaaattacaacgccgagcgataggtagacgaggagcaagaagacggcttgcctgatgatggaggtggagtc gattgtggtttggg

McK2P5-6.seq   GACCTGGGTAGTTGGGTTTGGATTGAGGTCTTGCATAACGGCCA                                                          944
MdK2P5.seq     GACCTGGGTAGTTGGGTTTGGATTGAGGTCTTGCATAACGGCCA                                                          944
Consensus      gacctgggtagttgggtttggattgaggtcttgcataacggcca
```

Supplementary Figure S6. Nucleotide alignments of *MsK2P5-6* with *MdK2P5* from NCBI. Comparison of nucleotide of *MsK2P5-6* we cloned and *MdK2P5* (LOC103405965) downloaded from NCBI. The similarity was 99.47%. NCBI, National Center for Biotechnology Information.

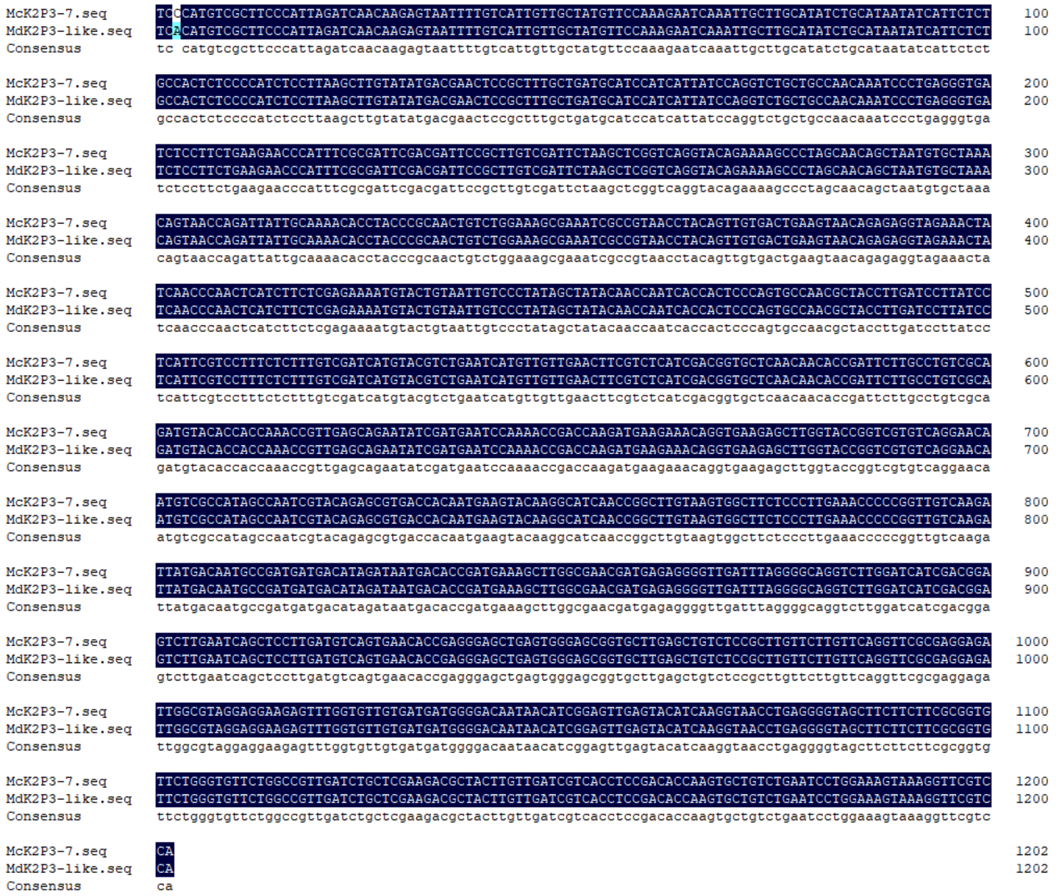

Supplementary Figure S7. Nucleotide alignments of *MsK2P3-7* with *MdK2P3-like* from NCBI. Comparison of nucleotide of *MsK2P3-7* we cloned and *MdK2P3-like* (LOC103443184) downloaded from NCBI. The similarity was 99.92%. NCBI, National Center for Biotechnology Information.

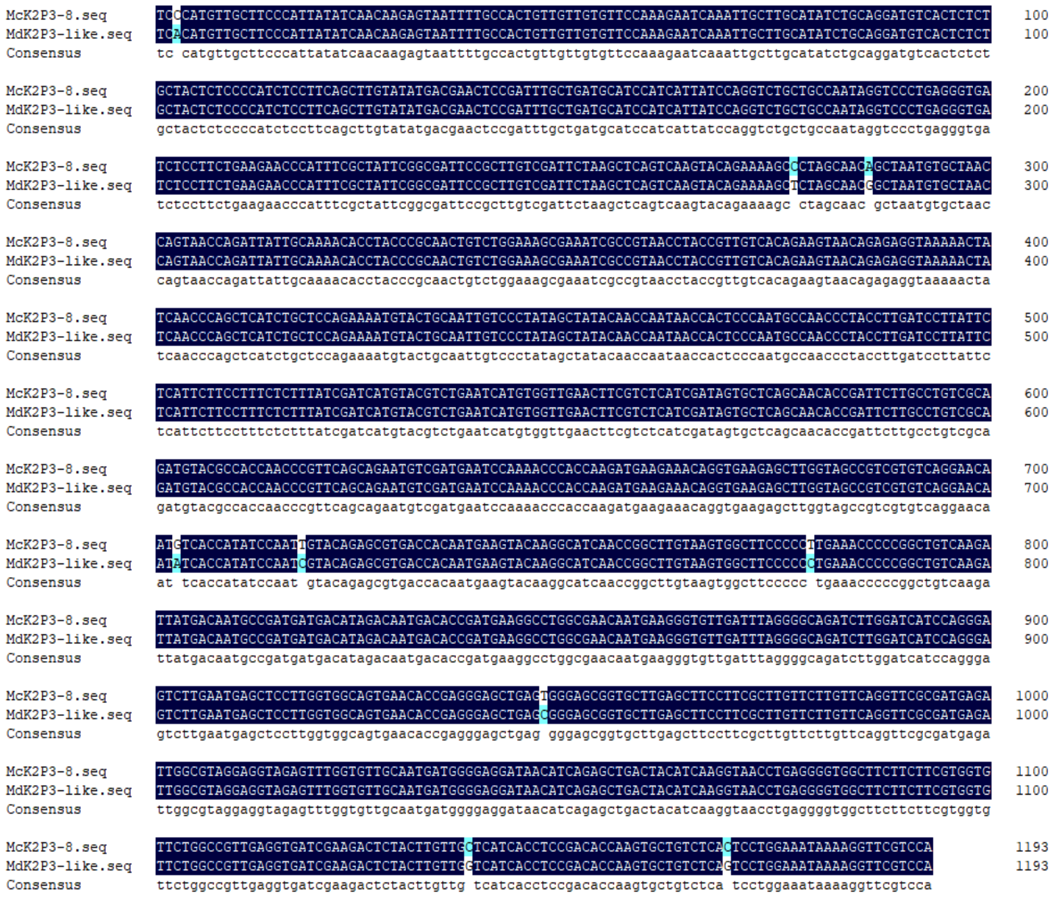

Supplementary Figure S8. Nucleotide alignments of *MsK2P3-8* with *MdK2P3-like* from NCBI. Comparison of nucleotide of *MsK2P3-8* we cloned and *MdK2P3-like* (LOC103405172) downloaded from NCBI. The similarity was 99.25%. NCBI, National Center for Biotechnology Information.

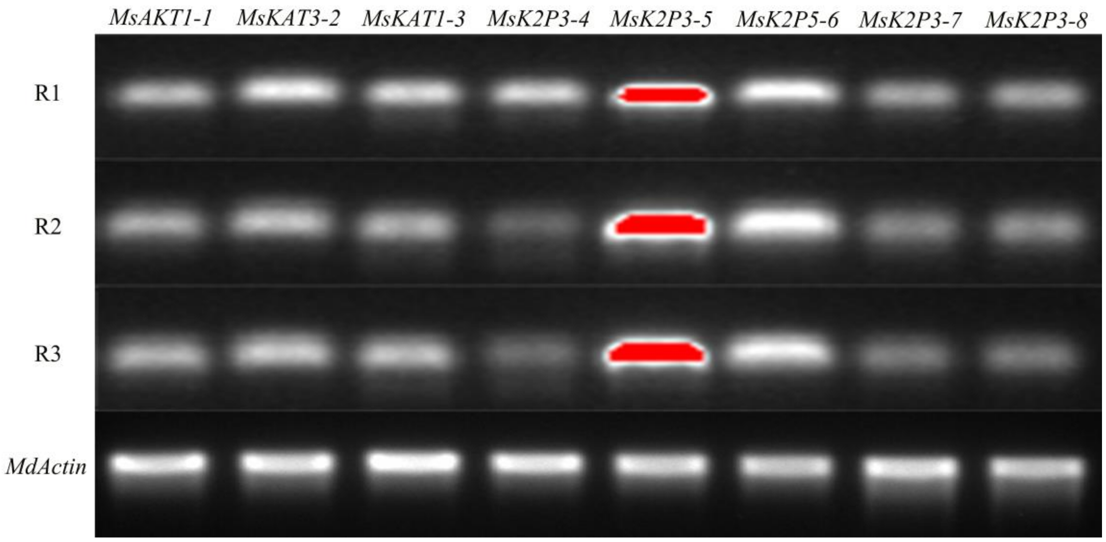

Supplementary Figure S9. The gel image of eight genes in semi-quantitative RT-PCR detection. The first to third rows (R1-R3) represented the three biological replicates of eight genes.
